# Cytotoxicity of Odorous Compounds from Poultry Manure

**DOI:** 10.3390/ijerph13111046

**Published:** 2016-10-26

**Authors:** Adriana Nowak, Katarzyna Matusiak, Sebastian Borowski, Tadeusz Bakuła, Sebastian Opaliński, Roman Kołacz, Beata Gutarowska

**Affiliations:** 1 Institute of Fermentation Technology and Microbiology, Lodz University of Technology, Wolczanska 171/173, 90-924 Lodz, Poland; katarzyna.matusiak@p.lodz.pl (K.M.); sebastian.borowski@p.lodz.pl (S.B.); beata.gutarowska@p.lodz.pl (B.G.); 2 Department of Veterinary Prevention and Feed Hygiene, University of Warmia and Mazury in Olsztyn, Oczapowskiego 13, 10-718 Olsztyn, Poland; bakta@uwm.edu.pl; 3 Department of Environment, Hygiene and Animal Welfare, Wroclaw University of Environmental and Life Sciences, Chelmonskiego 38 C, 51-630 Wroclaw, Poland; sebastian.opalinski@up.wroc.pl (S.O.); roman.kolacz@up.wroc.pl (R.K.)

**Keywords:** ammonium, dimethylamine, trimethylamine, poultry, LMH cells, cytotoxicity, MTT, IC_50_

## Abstract

Long-term exposure and inhalation of odorous compounds from poultry manure can be harmful to farm workers and the surrounding residents as well as animals. The aim of the present study was to determine the cytotoxicity and IC_50_ values of common odorous compounds such as ammonium, dimethylamine, trimethylamine, butyric acid, phenol, and indole in the chick liver hepatocellular carcinoma cell line LMH (*Leghorn Male Hepatoma*), in vitro, using MTT (3-(4,5-dimethylthiazolyl-2)-2,5-diphenyltetrazolium bromide) and PrestoBlue cytotoxicity assays. The cells were microscopically examined for any morphological changes post treatment. Dimethylamine exhibited the strongest cytotoxic effect on LMH cells with an IC_50_ value of 0.06% and 0.04% after an exposure of 24 h and 48 h, respectively. Both ammonium and trimethylamine had comparable cytotoxicity and their IC_50_ values were 0.08% and 0.04% after 24 h and 48 h, respectively. Of note, indole had the lowest cytotoxicity as the majority of cells were viable even after 72 h exposure. Thus, the IC_50_ for indole was not calculated. Results achieved from both MTT and PrestoBlue assays were comparable. Moreover, the morphological changes induced by the tested odours in LMH cells resulted in monolayer destruction, cytoplasm vacuolisation, chromatin condensation, and changes in nucleus and cell shape. Our study showed harmful effects of odorous compounds in chick tissues.

## 1. Introduction

Odorous compounds generated from poultry farmhouses are a potential nuisance to the environment and public health. Odours are formed by microbial decomposition of excreta from litter and direct emissions from the birds in a poultry farm [1]. They can originate from fresh and decomposing waste products such as manure and also from feathers and bedding. Strong odours have been reported to intensify the symptoms of people with asthma or allergies [2]. Odours may cause a variety of adverse reactions in people, such as emotional stress, headaches, acedia, insomnia, vomiting, irritation and depression. The public usually react to objectionable odorous episodes by registering complaints with the local authorities [3]. Animal feeding operations emit odour resulting from emissions of a large number of volatile compounds including ammonium, hydrogen sulphide, dimethylamine, trimethylamine, dimethyl sulphide, dimethyl trisulphide, 2,3-butanedione, 3-methyl-butanal, 1-butanol, 3-methyl-1-butanol, 3-hydroxy-2-butanone (acetoin), 2-butanone, phenol, cresol, acetic, propionic and butyric acids, indole, skatole, mercaptans (methyl-, ethyl-, propyl-), dimethyl disulphide and many others [4,5,6,7].

Of the several manure-based compounds, the most frequently reported compound is ammonium, which can cause health problems in both animals and human workers [8]. Ammonium is produced as a product of microbial decomposition of nitrogen containing organic compounds in the manure and hydrolysis of uric acid, which is favoured by pH ≥ 8. Evaporation of aqueous ammonium is the source of gaseous ammonium, which is a soluble and a reactive gas [9]. Gaseous ammonium can readily dissolve in water and react with other chemicals to form ammonium-containing compounds. The concentrations of ammonium in the air are greatest in areas where there is intensive livestock farming [10]. The production of bird odours is caused by an inappropriate diet with overabundance of amino acids and proteins. In practical poultry diets, approximately one-third of the nitrogen is incorporated into the tissues and eggs of the birds, and two-thirds is excreted [11]. It was suggested that ammonium should not exceed 25 ppm in poultry houses [11], however it was found that prolonged exposure to levels as low as 20 ppm compromises the immune system of chickens, making them more susceptible to diseases, bacterial infections—especially with *Escherichia coli*—and damage to the respiratory system [12]. Ammonium gas has a sharp and pungent odour and can act as an irritant when present in elevated concentrations [13]. High ammonium concentrations have an effect on chicken eyes causing inflammation of conjunctivae and damaging the cornea of the eyes [12]. Both concentration and exposure time may influence the effect of ammonium on both the poultry and the health of the farmers. It also decreases the egg production, food intake and body weight of chickens [14]. Symptoms of poisoning in poultry include tracheal irritation, air sac inflammation, conjunctivitis, dyspnoea and respiratory tract damage [11]. To date, there are limited published data on the mutagenicity of ammonium. One study on the mutagenic effect of ammonium noted an increase in chromosomal aberrations, sister chromatid exchanges and increased mitotic index in workers from a fertilizer factory [15,16]. Another study on poultry litter aqueous leachate, of which the main compound was ammonium, revealed its toxicity in an assay with *Ceriodaphnia dubia* as the test organism [17]. Aqueous leachate also showed toxicity in Microtox and *Daphnia* bioassays, as well as genotoxicity in the Ames test [18]. The global emission of ammonium and trimethylamine from animal husbandry is 23,300 Gg·N·year**^−^**^1^ and 108 Gg·N·year**^−^**^1^, respectively [19]. Moreover, aerobic biodegradation of uric acid and undigested animal fats and proteins in the litter also result in the formation of amines and volatile fatty acids [20].

Amine emissions of two to three orders of magnitude less than ammonia are also reported. Methylamine emissions are dangerous for the respiratory tract, as these may be the reason of short breath and sore throat. Dimethylamine is not classified as mutagenic or carcinogenic, but it may be toxic for the liver. Both may also lead to harmful changes in the lungs [21]. In addition, the irritant effects of dimethylamine on the respiratory epithelium cause reflex respiratory depression. The RD_50_ value (concentration which reduces the rate of respiration by 50%) after exposing laboratory mice to dimethylamine for 15 min was determined as 70 mL/m^3^ [22]. Meanwhile, no toxic effects of trimethylamine were reported in workers at mean exposure concentrations of 5 mL/m^3^ for 8 h, although concentrations of 20 mL/m^3^ were irritant to the mucous membranes and the eyes. Trimethylamine is formed during the decay of fish by bacterial decomposition. Those workers who were exposed to 940 mL/m^3^ and over 2000 mL/m^3^ faced eye problems including reddening, irritation and corneal clouding and also some central nervous system disturbances [23]. Studies in mice determined the RD_50_ for trimethylamine to be 61 mL/m^3^ [24]. Although di- and trimethylamines are classified as non-carcinogenic, after their uptake into the human (animal) body either by inhalation or direct contact, they can be converted to carcinogenic nitrosamines, such as *N*-nitrosodimethylamine, by a process called endogenous nitrosation [25]. These compounds can be formed by bacteria in the oral cavity and in the gastrointestinal tract of humans or animals. Moreover, acid-catalysed nitrosation is thought to be a possible mechanism for the in vivo formation of nitrosamines in the acidic environment of the stomach [26]. It was shown that dimethylamine nitrosation is enhanced in the presence of phenol [27]. Phenol and indole are products of degradation of aromatic amino acids (tyrosine, phenylalanine, tryptophan) descending from proteins [28]. Both compounds act like co-carcinogens as they enhance nitrosation of secondary amines. Additionally, indole is a known promoter of cancer [28,29]. The compound and its derivatives were also mutagenic in the Ames test [30]. Prolonged exposure to phenol induces necrosis of skin tissues and causes irritation of mucosal membranes. In addition to skin and mucosal membranes, the liver and cardiovascular system may also be targets for phenol toxicity [31]. Short-term exposure of mice to phenol caused respiratory irritation and reduction in respiratory rate (166 ppm reduced the respiratory rate by 50%). Studies in rats showed an increased incidence of a red nasal discharge, possibly due to the irritating properties of phenol (concentrations of up to 25 ppm for 2 weeks). Phenol also caused pneumonia, necrosis of the myocardium, liver and renal lesions in rabbits and guinea pigs. Inhaled phenol can affect numerous organs and tissues and can contribute to neurological effects [31]. In contrast, butyric acid can be the reason for burning sensations, cough, laboured breathing, shortness of breath, sore throat, eyes redness and pain, and loss of vision. Symptoms may be delayed with time [32].

There are no defined obligatory limits and legislation regulating odours (classification and emission) in Poland compared to many other European countries. Ammonium, in particular, has a number of guidelines and limitations to protect human health and safety in many European countries [33]. In Poland, ammonium emission is recommended not to exceed 20 ppm according to the Regulation of the Minister of Agriculture and Rural Development [34]. At present, the Directive 2010/75/EU of the European Parliament and the Council of 24 November 2010 on industrial emissions (integrated pollution prevention and control) is in force, setting limits for large combustion plants, cement kilns and cleaning of waste gases: mainly for carbon monoxide, sulphur dioxide and nitrogen dioxide. Thus all experimental data determining cytotoxic concentrations of these odours are very important.

The use of cell cultures in vitro is an alternative to predict acute lethality in vivo. There is still insufficient data associated with the cytotoxicity of poultry odours. There is no research on animal or human cell lines. The aim of the current study was to estimate the cytotoxicity (and IC_50_ values) of the most frequent odorous compounds from poultry manure (ammonium, dimethylamine, trimethylamine, indole, phenol and butyric acid) in a chick cell line model in vitro using two commercial cytotoxicity assays: MTT (3-(4,5-dimethylthiazolyl-2)-2,5-diphenyltetrazolium bromide) and PrestoBlue, together with microscopic observations of any morphological changes in the cells.

## 2. Materials and Methods

### 2.1. LMH (Leghorn Male Hepatoma) Cell Culture

Chicken liver hepatocellular carcinoma cell line LMH (CLS, Germany, lot no. 601411-714SF) from the 24th passage was used in this study. As the cells are adherent, they were cultured as a monolayer in collagen coated Roux flasks (BioCoat, Becton, Dickinson and Co., Franklin Lakes, NJ, USA) in Waymouyh’s Medium (Gibco, Thermo Fisher Scientific, Waltham, MA, USA) containing 7.5% sodium bicarbonate (Gibco, Thermo Fisher Scientific, Waltham, MA, USA), 10% heat-inactivated foetal bovine serum (FBS) (Gibco, Thermo Fisher Scientific, Waltham, MA, USA), 25 mM HEPES (Sigma-Aldrich, St. Louis, MO, USA), 100 IU/mL penicillin (Sigma-Aldrich, St. Louis, MO, USA), and 100 µg/mL streptomycin (Sigma-Aldrich, St. Louis, MO, USA). The cells were incubated in a CO_2_ incubator at 37 °C in 5% CO_2_ for 7 days to reach confluence. The medium was changed every 3–4 days. The cells were sub-cultivated after reaching confluence. They were detached with TrypLE^TM^ Express (Gibco, Thermo Fisher Scientific) for 5 min at 37 °C, suspended in sterile PBS (phosphate buffer saline) (Sigma-Aldrich, St. Louis, MO, USA), pH 7.2, and aspirated off the plastic flask. Due to the enzyme’s plant origin, the reaction did not require termination by addition of FBS. Following detachment, the cell suspension was transferred to a 15 mL Falcon tube, centrifuged at 182× *g* for 5 min, decanted and re-suspended in fresh medium. The cells were ready to use after cell count measurement and determination of viability by trypan blue exclusion of a minimum of 90%. Experiments with individual odour compounds and their time points were conducted with the same cell population.

### 2.2. Chemicals

Ammonium, dimethylamine, trimethylamine, indole, phenol and butyric acid were purchased from Sigma-Aldrich, St. Louis, MO, USA. The stocks were dissolved in Waymouyh’s Medium with no FBS and were sterile filtered using a 0.22 μM pore size filter (Membrane Solutions, Washington, DC, USA). The final tested concentrations for ammonium, dimethylamine and trimethylamine ranged from 0.004% to 1.0%. The concentration range was lower for phenol, indole and butyric acid (due to very low solubility in aqueous medium): 0.0004% to 0.1% for phenol; 0.0004% to 0.5% for indole; and 0.006% to 0.5% for butyric acid. All the stocks and their dilutions were freshly prepared on the day of experiment. These concentrations were determined on the basis of our previous work [7].

### 2.3. Cytotoxicity Testing

#### 2.3.1. MTT Assay

In the MTT assay, 3-(4,5-dimethylthiazol-2-yl)-2,5-diphenyltetrazolium bromide, a yellow tetrazolium is reduced to purple formazan in the mitochondria of living cells. The amount of formazan produced is proportional to the amount of MTT in the incubation medium.

For our experiment, 1 × 10^4^ LMH cells were placed in each well of a collagen coated 96-well plate (BioCoat, Becton, Dickinson and Co., Franklin Lakes, NJ, USA) and 100 µL of the complete culture medium was added into each well. The cells were incubated overnight at 37 °C in 5% CO_2_ to allow them to attach. The medium was aspirated the following day, and 200 µL of each concentration (see Materials and Methods Section 2.2) of the tested compound in Waymouyh’s Medium (Gibco, Thermo Fisher Scientific, Waltham, MA, USA) without FBS was added to each well in eight repeats. The control samples consisted of cells without the tested agent. Cells were incubated in a CO_2_ incubator at 37 °C in 5% CO_2_ for 24 h, 48 h and/or 72 h, depending on the odour tested.

After incubation, the medium with tested compounds was gently aspirated from each well and 100 µL of MTT (0.5 mg/mL in PBS, pH 7.2) was added and incubated at 37 °C in 5% CO_2_ for 3 h. MTT was then carefully removed and formazan precipitates were solubilised by adding 50 µL of DMSO (Sigma-Aldrich, St. Louis, MO, USA). Absorbance was measured at 550 nm with a reference filter of 620 nm, using a microplate reader (TriStar^2^ LB 942, Berthold Technologies GmbH and Co. KG, Bad Wildbad, Germany). The absorbance of the control sample (untreated cells) represented 100% cell viability. Cell viability (%) was calculated as follows: (sample OD/control OD) × 100%; and cytotoxicity (%) as: 100 − cell viability (%). Results were presented as mean ± standard deviation (SD). The mean error of the method is up to 10%.

#### 2.3.2. PrestoBlue Assay

PrestoBlue Cell Viability Reagent (Invitrogen, Thermo Fisher Scientific, Waltham, MA, USA) is used for rapid evaluation of the viability and proliferation of cells in a culture. It is a resazurin-based membrane permeable solution, which, upon reduction, forms a red fluorescent compound called resorufin using mitochondrial enzymes of the viable cells in the tested systems. As a consequence, the reagent exhibits a change in colour, as well as a shift in its fluorescence. Since ammonium, di- and tri-methylamine appeared to be the most cytotoxic to LMH cells in the MTT assay, these compounds were selected for analysis by PrestoBlue testing.

The experimental procedure for this assay was the same as MTT, except that special collagen coated, black 96-well plates for fluorescence were used (BioCoat, Becton, Dickinson and Co., Franklin Lakes, NJ, USA), and the cells were incubated with odours for 24 h. Following incubation, the medium with tested compounds was gently aspirated from each well and 100 μL of PrestoBlue reagent (10% solution in PBS, pH 7.2) was added to each well of the 96-well plate and incubated at 37 °C in 5% CO_2_ for 2 h, as recommended. Since fluorescence is more sensitive than absorbance in this assay, it is the recommended detection method [30]. Fluorescence was measured at 560 nm excitation and 590 nm emission, using a microplate reader. Cell viability (%) was calculated as described earlier. Experiments with these compounds was conducted with the same cell population. Results were presented as mean ± standard error of the mean (S.E.M).

### 2.4. Estimation of IC_50_

The values of IC_50_, which is the concentration of the test compound required to reduce the cell survival fraction to 50% of the control, were used as a measure of cellular sensitivity to a given treatment. IC_50_ values were read from dose-response curves and determined according to the formula: IC_50_ = (X − Z)/(X − X_1_) × V(C_X1_ − C_X_) + C_X_, where X is 50% decrease in viability; Z is % of viability >Z; X_1_ is % viability <Z; C_X_ is concentration of the compound for X and C_X1_ is concentration of the compound for X_1_ [35].

### 2.5. Microscopic Observations of Morphologic Changes

Morphological changes of LMH cells in the presence of tested compounds were observed in 4-well Lab-Tek^TM^ Chamber Slides (Nunc, Thermo Fisher Scientific, Waltham, MA, USA). The slides were coated with collagen I (Gibco, Thermo Fisher Scientific, Waltham, MA, USA) according to the manufacturer’s instructions. LMH cells were seeded on each well by adding 2.5 × 10^5^ cells/well. Cells were treated the same way as the MTT assay. The influence of the chosen concentrations of odour compounds were checked after 24 h incubation. Living cells were observed with an inverted microscope (Delta Optical IB-100, Nowe Osiny, Mińsk Mazowiecki, Poland) connected to a digital camera (HDCE-X5, Nowe Osiny, Mińsk Mazowiecki, Poland) and the imaging software ScopeImage 9.0 (Nowe Osiny, Mińsk Mazowiecki, Poland). After 24 h exposure, the medium with the compounds was gently aspirated, cells were washed with PBS (pH 7.2) and fixed with 500 μL of 70% ethanol for 10 min at room temperature. The cells were then stained with the Giemsa/May-Grünwald (Sigma-Aldrich, St. Louis, MO, USA) method. After staining, the wells were washed with 70% ethanol until no colour remained and air-dried. The morphology of LMH cells was observed at 1000× magnification under a phase-contrast microscope (Nikon, Tokyo, Japan) connected to a digital camera (Nikon Digital Sight DS-U3, Tokyo, Japan) and an imaging software (NIS-elements BR 3.0, Nikon, Tokyo, Japan).

### 2.6. Statistical Analysis

Two-way analysis of variance (ANOVA) was conducted using OriginPro 6.1 (Northampton, MA, USA) software to evaluate the experimental data. The significant differences between the means were compared using Scheffe’s multiple comparison test and they were accepted to be significant at *p* < 0.05 (Statistica 10, StatSoft, Tulsa, OK, USA).

## 3. Results

### 3.1. Assessment of the Cytotoxicity of Odours

#### 3.1.1. By MTT Assay

**Figure 1:**
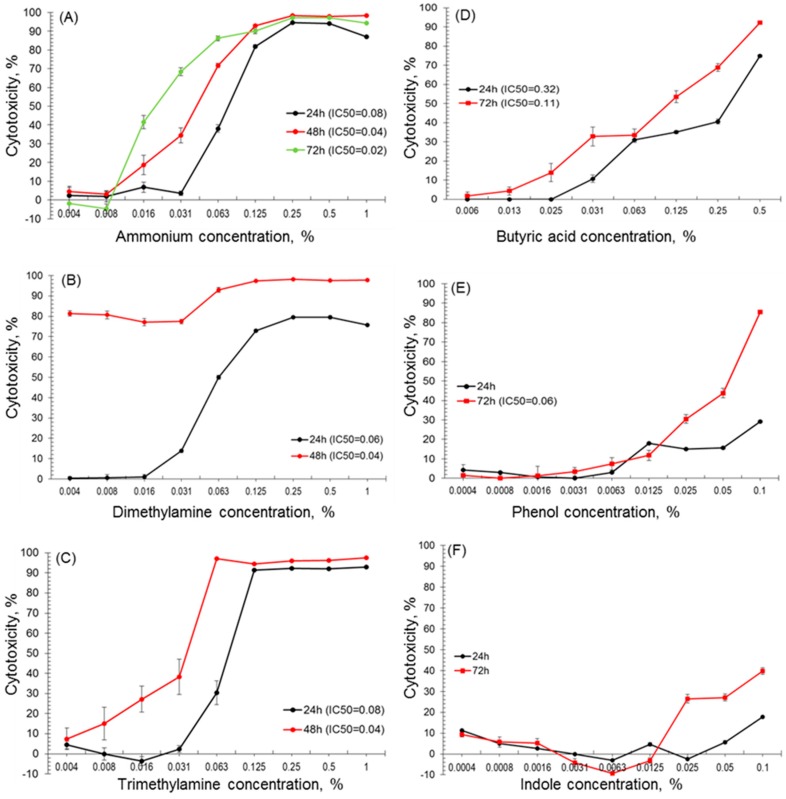
Cytotoxic activity of odorous compounds: (**A**) ammonium; (**B**) dimethylamine; (**C**) trimethylamine; (**D**) butyric acid; (**E**) phenol and (**F**) indole measured by the MTT (3-(4,5-dimethylthiazolyl-2)-2,5-diphenyltetrazolium bromide) assay in the LMH (*Leghorn Male Hepatoma*) chicken cell line after 24 h, 48 h and/or 72 h exposure. Each data point represents the mean of the absorbance values from cells from eight individual wells (±SD—standard deviation).

The LMH cells were challenged with the compounds over a range of concentrations from 0.004% to 1% for ammonium, di- and trimethylamine; 0.006% to 0.5% for butyric acid and 0.0004% to 0.1% for phenol and indole. Cell proliferative activity and viability was measured by the colorimetric MTT assay after 24 h, 48 h and/or 72 h, in eight repeats for each concentration. Viability of cells and statistical significance (ANOVA, *p* < 0.05) was calculated and is shown in Table 1. The dependence between odour concentration and its cytotoxicity (anti-proliferative activity) is graphically presented (Figure 1).

In the presence of dimethylamine, the viability of LMH cells proportionally decreased with increasing concentration of the compound (Table 1). Cell viability in the presence of the highest test concentration (1%) of dimethylamine was up to 24.4% ± 0.2% (*p* < 0.05) after 24 h. In contrast, cell viability was much lower in the presence of the same concentration of ammonium and trimethylamine at 13.1% ± 0.1% and 7.0% ± 0.1%, respectively. However, after 48 h, the viability highly decreased to 18.6% even in the presence of the lowest concentration of dimethylamine 0.004% (*p* < 0.05, Table 1). We observed that cell viability started to decrease significantly from 0.063% concentration of ammonium after 24 h exposure (61.9% ± 2.0%) and in the presence of 1% it was only 13.1% ± 0.1% (Table 1). After 48 h and 72 h, a strong decrease in cell viability was observed for cells treated with a 0.016% concentration of ammonium, and all results from that concentration were statistically significant in comparison to the control (*p* < 0.05, Table 1). Butyric acid, after 72 h incubation, reduced cell viability to 7.6% ± 0.7% in the presence of the highest tested concentration (0.5%) (*p* < 0.05, Table 1). LMH cells preserved quite high viability after 72 h exposure to 0.1% of indole, and were measured as 60.3% ± 1.6% (*p* < 0.05, Table 1).

As ammonium is produced in poultry farmhouses in very high levels, exposure of LMH cells to the odour was prolonged to 72 h. Its cytotoxicity definitely increased from a 0.016% concentration (41.5% ± 3.5%) (Figure 1A). Dimethylamine displayed the strongest cytotoxic (anti-proliferative) effect toward LMH cells (Figure 1B). After 48 h exposure, all concentrations of dimethylamine induced cytotoxicity above 80%, while in the case of trimethylamine the cytotoxicity increased significantly and exposure to a 0.063% concentration resulted in cytotoxicity above 95% (Figure 1C). The cytotoxicity of butyric acid was increasing along with a growing concentration and after 72 h it was 92.4% ± 0.7% after exposure to 0.5% butyric acid (Figure 1D). The lowest cytotoxicity was observed for indole and phenol (Figure 1E,F). These compounds were not cytotoxic after 24 h exposure. After 72 h, concentrations above 0.0125% caused a dose-dependent inhibition. Indole either slightly stimulated or suppressed the proliferation of LMH cells. After 72 h, the highest tested concentration of indole (0.1%) demonstrated 39.7% ± 1.6% cytotoxicity, while in the case of phenol (0.1% after 72 h) it was two-fold higher at 85.5% ± 0.8%.

#### 3.1.2. By PrestoBlue Assay

**Figure 2:**
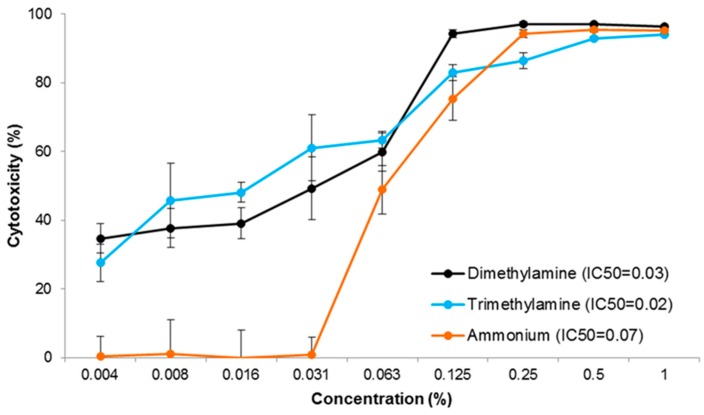
Cytotoxic activity of odorous compounds (ammonium, di- and trimethylamine) estimated by the PrestoBlue assay in the LMH chicken cell line after 24 h exposure. Each data point represents the mean of the fluorescence values of cells from eight individual wells (±S.E.M.).

Ammonium, di- and trimethylamine caused the highest cytotoxicity in LMH cells as seen in the MTT assay. These compounds were therefore chosen for PrestoBlue testing. Viability of the cells and statistical significance (ANOVA, *p* < 0.05) was calculated and is shown in Table 2. The dependence between odour concentration and its cytotoxicity is graphically presented in Figure 2.

The LMH cells were challenged with the compounds for 24 h with concentrations ranging from 0.004% to 1.0% in eight repeats for each concentration. Ammonium significantly (*p* < 0.05) reduced cell viability at a concentration of 0.063% and in the presence of 1% ammonium, the viability further reduced to 4.7% ± 0.7% (Table 2). Both di- and trimethylamine at a concentration of 1%, highly reduced cell viability to 3.7% ± 0.5% and 6.0% ± 0.3%, respectively (*p* < 0.05, Table 2). Both amines showed higher cytotoxicity than ammonium in the concentration range of 0.004% to 0.031% (Figure 2). For concentrations above 0.063%, the cytotoxicity of all compounds increased correspondingly and it reached 95% in the presence of 1% of each tested odour (Figure 2).

### 3.2. IC_50_ Calculations

The IC_50_ values of odorous compounds estimated in MTT and PrestoBlue assays in the LMH chick cell line were calculated from the dose-response curves (Table 3 and Table 4).

Dimethylamine appeared to be the most cytotoxic of all tested compounds. In the MTT assay, the IC_50_ after 24 h exposure was 0.06%, but after 48 h it was not possible to extrapolate the value from the dose-response curve due to the very high cytotoxicity of dimethylamine (Table 3). Thus, the IC_80_ value was calculated at 0.04% indicating the concentration of dimethylamine required to reduce the cell survival fraction by 80% in relation to the control. Ammonium and trimethylamine appeared to be the second most toxic compounds and their IC_50_ values were at the same level after both time points: 0.08% and 0.04% after 24 h and 48 h, respectively (Table 3). In the PrestoBlue assay, comparable values of IC_50_ were calculated for di- and trimethylamine after 24 h exposure of chick LMH cells at 0.03% and 0.02%, respectively. For ammonium it was at similar levels as determined by the MTT assay at 0.07% (Table 4).

Butyric acid had moderate cytotoxicity in LMH cells and after 72 h the IC_50_ of butyric acid was 0.11% (Table 3). Indole was the least toxic in LMH cells after 24 and 72 h exposure and it was impossible to determine its IC_50_ from our experimental settings which included poor solubility in aqueous incubation medium. The same situation was observed for phenol after 24 h exposure. After prolonged incubation of up to 72 h, the IC_50_ value for indole was equal to 0.06% (Table 3).

### 3.3. Morphology of LMH Cells

**Figure 3:**
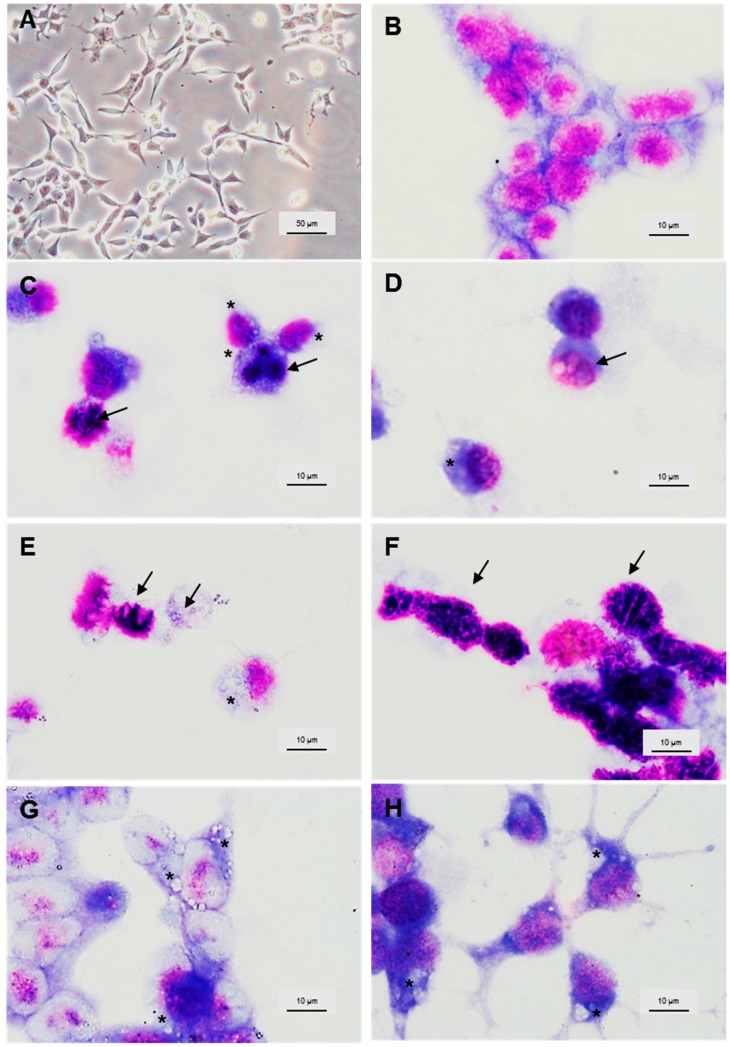
Representative micrographs showing morphology of LMH cells after 24 h incubation with the tested poultry odorants. (**A**) live cells at 200× magnification; (**B**–**H**) cells fixed and stained with Giemsa/May-Grünwald and visualised at 1000× in immersion oil. (**A**,**B**) untreated control; (**C**) multi-lobed nucleus and (**D**) nuclear abnormalities after treatment with ammonium (0.063%) as indicated by arrows; (**E**) anuclear cell and chromatin condensation after exposure to dimethylamine (0.063%) as indicated by arrows; (**F**) chromatin condensation after treatment with trimethylamine (0.063%) as indicated by arrows; (**G**) vacuolisation and swelling of cells upon exposure to butyric acid (0.25%); (**H**) elongated appendages after incubation with indole (0.1%). Asterisks indicate vacuoles.

Morphological alterations in LMH cells after exposure to the chemicals used in this study were qualitatively investigated using a light phase contrast microscope. Typically, LMH cells are medium-sized, dendritic-like adherent cells growing as a confluent monolayer. In our research, the untreated control cells were homogeneously distributed in the culture field (Figure 3A,B). The impact on cell morphology was visualised after 24 h exposure with each compound. The reduction in cell density and the number of detached and floating cells were observed. The number of cells per visual field appeared fewer than the untreated control, and the monolayer was no longer confluent. Also, the cells were swollen and had lost their typical dendritic shape (Figure 3C–H). In the presence of 0.063% ammonium (Figure 3C,D), chromatin condensation, as well as multi-lobed and nuclear abnormalities were observed. Shrinking of the cytoplasm, loss of cell membrane, chromatin condensation and increased vacuolisation occurred after incubation with 0.063% DMA and TMA (Figure 3E,F). Butyric acid (0.25%) induced detachment of cells from their neighbours, vacuoles, cytoplasmic granules condensation and swelling of the cells (Figure 3G). Vacuolisation of the cytoplasm, loss of shape and elongation of cell appendages were typical changes in the presence of indole and phenol (0.1%) (Figure 3H).

## 4. Discussion

The consumption of poultry and poultry products is increasing all over the world. This growth has resulted in an increase in associated waste, which, in turn, correlates with an increase in the level of odours emitted. Odours can be harmful to farm workers and surrounding residents, and may also cause irritation of the respiratory tract in humans and animals with long-term exposure.

In our research, the cytotoxic activity of six prevalent odoriferous compounds produced from poultry manure were investigated. Three of them, namely ammonium, di- and trimethylamine, were the most cytotoxic in both MTT and PrestoBlue assays. Generally, both assays showed a dose-dependent response with ammonium, di- and trimethylamine toxicity. In our study, the sub-toxic doses of ammonium (i.e., 0.004% and 0.008% after 72 h incubation), trimethylamine (i.e., 0.016% after 24 h incubation) and indole (i.e., 0.0031%–0.0125% after 24 and 72 h incubation) only slightly stimulated cell proliferation and viability. However, at higher concentrations, a dose-dependent cytotoxic effect was found. This effect is typical for many cytotoxic drugs and anticancer agents and can be interpreted as a rescue mechanism of the exposed cells [36]. In MTT assay, a minimum of 1000 cells per well is mandatory in comparison to PrestoBlue, which, according to Boncler et al. [37] and Xu et al. [38] requires only 12 cells per well. Therefore, PrestoBlue is a more sensitive assay than MTT. Both assays gave similar results in evaluating the IC_50_ values although results achieved with PrestoBlue should be treated as more accurate.

There are few in vitro studies connected with the cytotoxicity of poultry odours towards animal cells. Olejnik et al. [39] evaluated the cytotoxicity of phenol and ammonium in terms of influence on survival and proliferation of the human colon adenocarcinoma cell line Caco-2, as these are compounds result from protein digestion in the colon. They showed that than even at concentrations as low as 10**^−^**^6^ M, these compounds reduced cell proliferation and cell survival to 56%. Lestari et al. [40], using the MTS (3-(4,5-dimethylthiazolyl-2)-2,5-diphenyltetrazolium bromide) assay, examined the cytotoxicity of ammonium in three cell lines: human hepatocarcinoma HepG2, human skin fibroblasts and human epithelial lung carcinoma cell line A549. The IC_50_ values for ammonium were 17.4 mM, 9.4 mM and 15.4 mM, respectively, which correspond to 0.04%, 0.03% and 0.02% values. In our study with the chicken LMH cell line, the IC_50_ for ammonium was 0.08% and 0.07% in MTT and PrestoBlue assays, respectively. In studies by Mouillé et al. [41], 20 mM (or 0.04%) of ammonium almost suppressed proliferation of the human adenocarcinoma cell line HT-29 cell, but did not increase the amount of floating cells or the amount of necrotic cells to any significant extent when compared with the control untreated cells. The chick LMH cells appear to be more resistant to the compound compared to the human Caco-2 and HT-29 cells.

In our research, dimethylamine was the most toxic, because all the nine tested concentrations significantly reduced cell viability after 48 h exposure when compared to the medium control (*p* < 0.05). Indole was the least cytotoxic compound—its IC_50_ value was not likely to be determined during 72 h investigation.

Butyric acid is a volatile component of decomposing manure, while fresh manure is found to be devoid of this component [42]. The compound appeared to be less toxic in LMH cells in comparison to ammonium or the tested methylamines. It seemed to be more cytotoxic after prolonged exposure (after 72 h). It depressed proliferation of LMH cells with increasing concentration. After 72 h exposure, concentrations above 0.0125% caused a dose-dependent inhibition, reaching 92% (*p* < 0.05). It is in accordance with the investigation done by Kurita-Ochiai et al. [43]. In the Jurkat human T-lymphoma cell line, they observed the same dependence for concentrations above 0.02% in the MTT assay. The authors concluded that the antiproliferative effect of butyric acid in human Jurkat cells is mediated through the induction of apoptotic cell death. Furthermore, the cytotoxic activity of butyric acid can be a reason of acidification of the culture medium. In case of ammonium, di- and trimethylamine, the culture medium can be affected by its alkalisation. Our findings may suggest the use of in vitro cytotoxicity assays as good models for predicting the toxicity of substances which have acid and alkaline properties.

The cytotoxic effect of the tested odours was supported by microscopic observations of morphological changes in LMH cells after 24 h treatment. Exposure to the tested compounds resulted in monolayer destruction; high levels of cytoplasm vacuolisation (presence of autophagosomes and autolysosomes); some of the compounds caused nuclear chromatin condensation in the majority of cells; karyolysis (presence of anuclear cells); and changes in nucleus and cell shape. The main mechanism of cytotoxicity induced by odours seems to be cell injury leading to its detachment.

## 5. Conclusions

Our data clearly demonstrated marked cytotoxic effects and acute cell damage produced by odours from poultry manure. IC_50_ values for tested compounds seem to be low, so their cytotoxic activity in the chick LMH cell line is high. Of note, odours formed in breeding farms are a complex mixture of gases, made up of over 160 chemical components [44], and their harmful effects can be really fortified in vivo. Correlation between in vitro cytotoxicity data and in vivo animal and human toxicity must be supported by further studies. The abatement of odour emissions from poultry houses can be achieved by prevention and treatment methods (both biological and chemical) and should be given the highest level of priority.

## Figures and Tables

**Table 1:** Viability of chicken LMH hepatocytes in the presence of odorous compounds from poultry manure determined by the MTT assay. Each value represents the mean of eight repeats. * indicates results significantly different from the control sample at appropriate time-points; ANOVA (*p* < 0.05).

Odour Compound	Concentration (%)	Viability of LMH Cells (%) ± SD		
		24 h	48 h	72 h
Ammonium	0.004	97.6 ± 4.3	95.6 ± 2.9	101.9 ± 5.0
	0.008	98.0 ± 3.0	96.8 ± 1.3	104.8 ± 2.6
	0.016	93.3 ± 2.8	81.4 ± 5.2 *	58.5 ± 3.5 *
	0.031	96.4 ± 1.2	65.5 ± 4.0 *	31.6 ± 2.1 *
	0.063	61.9 ± 2.0 *	28.3 ± 1.3 *	13.8 ± 1.2 *
	0.125	18.1 ± 0.8 *	7.1 ± 0.8 *	10.0 ± 1.3 *
	0.25	5.4 ± 0.1 *	1.6 ± 0.2 *	2.9 ± 0.1 *
	0.5	5.9 ± 0.2 *	2.1 ± 0.2 *	2.8 ± 0.2 *
	1.0	13.1 ± 0.1 *	1.7 ± 0.2 *	5.8 ± 0.1 *
Dimethylamine	0.004	99.6 ± 0.5	18.6 ± 1.4 *	not determined
	0.008	99.3 ± 0.7	19.3 ± 1.9 *	
	0.016	99.0 ± 0.8	22.9 ± 1.9 *	
	0.031	86.2 ± 1.0	22.6 ± 1.1 *	
	0.063	50.0 ± 0.7 *	7.0 ± 1.2 *	
	0.125	27.2 ± 0.2 *	2.6 ± 0.2 *	
	0.25	20.5 ± 0.1 *	1.9 ± 0.2 *	
	0.5	20.5 ± 0.1 *	2.4 ± 0.1 *	
	1.0	24.4 ± 0.2 *	2.2 ± 0.1 *	
Trimethylamine	0.004	95.5 ± 1.9	92.5 ± 5.5	not determined
	0.008	100.1 ± 3.1	84.9 ± 8.1	
	0.016	103.6 ± 2.2	72.8 ± 6.5 *	
	0.031	97.8 ± 1.8	61.7 ± 8.9 *	
	0.063	69.7 ± 5.9 *	2.8 ± 0.2 *	
	0.125	8.6 ± 0.5 *	5.4 ± 0.3 *	
	0.25	7.6 ± 0.3 *	4.1 ± 0.3 *	
	0.5	7.9 ± 0.2 *	3.8 ± 0.3 *	
	1.0	7.0 ± 0.1 *	2.5 ± 0.3 *	
Butyric acid	0.006	100.0 ± 0.7	not determined	98.2 ± 2.1
	0.013	100.0 ± 0.4		95.7 ± 2.1
	0.025	100.0 ± 0.3		86.0 ± 4.8
	0.031	89.3 ± 2.0		67.2 ± 4.9 *
	0.063	68.9 ± 1.4 *		66.5 ±3.2 *
	0.125	65.0 ± 0.8 *		46.4 ± 3.2 *
	0.25	59.4 ± 1.3 *		31.1 ± 2.0 *
	0.5	25.1 ± 0.7 *		7.6 ± 0.7 *
Phenol	0.0004	95.8 ± 0.4	not determined	98.5 ± 5.5
	0.0008	96.9 ± 0.4		100.5 ± 1.7
	0.0016	99.4 ± 0.3		98.7 ± 4.8
	0.0031	100.0 ± 0.4		96.6 ± 2.3
	0.0063	96.9 ± 0.3		92.5 ± 3.1
	0.0125	82.1 ± 0.3 *		88.2 ± 2.6 *
	0.025	85.0 ± 0.2 *		69.5 ± 2.2 *
	0.05	84.4 ± 0.5		56.2 ± 2.4 *
	0.1	70.8 ± 0.5 *		14.5 ± 0.8 *
Indole	0.0004	88.6 ± 0.6	not determined	90.7 ± 1.3
	0.0008	95.0 ± 0.2		94.3 ± 2.4
	0.0016	97.4 ± 0.2		94.8 ± 2.1
	0.0031	100.0 ± 0.4		104.1 ± 1.9
	0.0063	103.0 ± 0.4		109.3 ± 2.5
	0.0125	95.5 ± 0.8		103.2 ± 1.2
	0.025	102.4 ± 0.2		73.5 ± 2.0 *
	0.05	94.3 ± 0.6		73.0 ± 1.7 *
	0.1	82.2 ± 0.2 *		60.3 ± 1.6 *

**Table 2:** Viability of chicken LMH hepatocytes in the presence of odorous compounds from poultry manure determined by the PrestoBlue assay after 24 h exposure. Each value represents the mean of eight repeats (±S.E.M.). * indicates results significantly different from the control sample at appropriate time-points; ANOVA (*p* < 0.05).

Concentration (%)	Viability of LMH Cells (%) ±S.E.M.		
	Ammonium	Dimethylamine	Trimethylamine
0.004	99.7 ± 5.8	65.3 ± 4.2	72.4 ± 5.4
0.008	99.0 ± 10.1	62.3 ± 5.6	54.3 ± 10.9
0.016	100.2 ± 8.1	60.9 ± 4.5	51.9 ± 2.9
0.031	99.1 ± 4.9	50.7 ± 9.1	38.9 ± 9.6
0.063	51.1 ± 7.1 *	40.2 ± 5.6	36.6 ± 2.4
0.125	24.6 ± 6.3 *	5.7 ± 1.2 *	17.1 ± 2.3 *
0.25	5.7 ± 1.1 *	2.8 ± 0.5 *	13.6 ± 2.3 *
0.5	4.6 ± 0.6 *	3.0 ± 0.5 *	7.1 ± 0.4 *
1.0	4.7 ± 0.7 *	3.7 ± 0.5 *	6.0 ± 0.3 *

**Table 3:** The IC_50_ values of odorous compounds estimated by the MTT assay in the LMH chicken cell line calculated from the dose-response curves.

Odour	IC_50_ (%)		
	24 h	48 h	72 h
Ammonium	0.08	0.04	0.02
Dimethylamine	0.06	0.04 *	not determined
Trimethylamine	0.08	0.04	not determined
Butyric acid	0.32	not determined	0.11
Phenol	not detected	not determined	not detected
Indole	not detected	not determined	0.06

* IC_80_ for dimethylamine after 48 h exposure.

**Table 4:** The IC_50_ values of odorous compounds estimated by the PrestoBlue assay in the LMH chicken cell line after 24 h exposure calculated from the dose-response curves.

Odour	IC_50_ (%)
Ammonium	0.07
Dimethylamine	0.03
Trimethylamine	0.02
